# Terminal restriction fragment length polymorphism is an “old school” reliable technique for swift microbial community screening in anaerobic digestion

**DOI:** 10.1038/s41598-018-34921-7

**Published:** 2018-11-14

**Authors:** Jo De Vrieze, Umer Z. Ijaz, Aaron M. Saunders, Susanne Theuerl

**Affiliations:** 1 Center for Microbial Ecology and Technology (CMET), Ghent University, Coupure Links 653, B-9000 Gent, Belgium; 2 Infrastructure and Environment Research Division, School of Engineering, University of Glasgow, Glasgow, UK; 3 Department of Biotechnology, Chemistry and Environmental Engineering, Aalborg University, Sohngardsholmsvej 49, 9000 Aalborg, Denmark; 4 Leibniz Institute for Agricultural Engineering and Bioeconomy e.V. (ATB), Department Bioengineering, Max-Eyth-Allee 100, D-14469 Potsdam, Germany

## Abstract

The microbial community in anaerobic digestion has been analysed through microbial fingerprinting techniques, such as terminal restriction fragment length polymorphism (TRFLP), for decades. In the last decade, high-throughput 16S rRNA gene amplicon sequencing has replaced these techniques, but the time-consuming and complex nature of high-throughput techniques is a potential bottleneck for full-scale anaerobic digestion application, when monitoring community dynamics. Here, the bacterial and archaeal TRFLP profiles were compared with 16S rRNA gene amplicon profiles (Illumina platform) of 25 full-scale anaerobic digestion plants. The α-diversity analysis revealed a higher richness based on Illumina data, compared with the TRFLP data. This coincided with a clear difference in community organisation, Pareto distribution, and co-occurrence network statistics, *i.e*., betweenness centrality and normalised degree. The β-diversity analysis showed a similar clustering profile for the Illumina, bacterial TRFLP and archaeal TRFLP data, based on different distance measures and independent of phylogenetic identification, with pH and temperature as the two key operational parameters determining microbial community composition. The combined knowledge of temporal dynamics and projected clustering in the β-diversity profile, based on the TRFLP data, distinctly showed that TRFLP is a reliable technique for swift microbial community dynamics screening in full-scale anaerobic digestion plants.

## Introduction

Anaerobic digestion (AD) can be considered the first microbial technologies for organic waste treatment to reach full-scale application, and it has been implemented extensively treating various waste streams since the 1980s^1–3^. Its widespread application is the consequence of (1) its ability to combine organic waste treatment with energy recovery, compared to other treatment technologies, and (2) the potential for process monitoring through, if desirable, on-line monitoring of biogas yield and composition, pH, alkalinity and volatile fatty acids (VFA) concentrations^4,5^. The appearance of new biomass streams, due to the integration of AD into bioeconomic production systems for food, feed, bioenergy and biomaterials, especially due to the need for energy-neutral and renewable production processes, enforces the efforts to maximize AD process efficiency^6,7^. This requires much finer control of the AD process, due to its complex character, to ensure the central role of AD in the bio-based economy^8–10^. The development of alternative process control strategies and early-warning indicators for instabilities enables more direct process control^11–14^. Nonetheless these indicators do not directly reflect microbial community dynamics and functioning.

In the last decades, simple but robust culture-independent techniques, such as denaturing gradient gel electrophoresis^15–17^, fluorescent *in situ* hybridisation^18,19^ and terminal restriction length polymorphism (TRFLP)^20–22^ have been used to study the microbial community organisation in AD. Recently, more powerful tools have become available that enable to generate detailed information about the genomic structure and gene expression, which reflects the putative and actual microbial metabolism, but also allow for analysing complex environments and their harbouring microbiomes^23–28^. The application of 16S rRNA gene amplicon sequencing to unravel the microbial community “black box” led to numerous discoveries of previously unknown microbial life, *e.g*., in AD. The clear description of the different AD process stages^29^, as well as the identification of the key species involved in the different steps of the process^30–32^ served as a general basis for process engineering. The overall sensitivity of the methanogenic community and the clear difference in metabolic and physiologic properties among different methanogens denoted methanogenesis as one of the crucial step**s** with respect to process control^33,34^. Higher microbial community dynamics were observed in response to changing conditions and/or (partial) inhibition of methanogenesis^35–39^. Such information can be used to relate microbial community dynamics with AD functional stability to predict and anticipate to process failure^40,41^, and to determine the main factors that drive the microbial community composition and organization^42^.

The application of next generation amplicon sequencing to make an estimation of microbial community stability in full-scale digesters poses issues in terms of (1) the operational (consumables) and capital (hardware) costs, and (2) the complexity and computationally demanding nature of data analysis. Especially the complexity of the data analysis is an important bottleneck with respect to full-scale application, as robustness and simplicity are considered crucial for frequent and fast application^8^. For fast and simple screening of the microbial community, the extra expense of amplicon sequencing, compared with TRFLP, may be unnecessary^23,43,44^. The key issue is that simple fingerprinting methods, such as TRFLP, may still capture the important population changes that are critical for process control. The key drawback of TRFLP analysis, *i.e*., the limited phylogenetic identification depth, due to the short read lengths following the restriction reaction^43^, does not prevent the determination of microbial community diversity and organisation through various methods^45^. Comparison of TRFLP with next generation amplicon sequencing based on the 16S rRNA gene to screen the microbial community showed the potential of TRFLP for “fast” community screening of the nasopharyngeal microbial community^46^. Comparable results between TRFLP and next generation sequencing methods (*via* the 454 and Illumina and Ion Torrent platforms) were also obtained for the microbial community in the rumen of sheep^47,48^, the gut of broiler chickens^49^, polar soils^50^, and aquifers^51^. The combination of next generation amplicon sequencing and TRFLP analysis in AD ecosystems, thus far, remained restricted to lab-scale experiments^52,53^ or only a limited number of full-scale plants^54^. Hence, even though these studies yielded interesting results, the observed differences between the TRFLP and next generation amplicon sequencing microbial community profile in AD requires further investigation. This will allow to determine the different levels of community profiling for which TRFLP can serve as *a proxy* of next generation amplicon sequencing in AD.

The objective of this research was to investigate the microbial community in various full-scale AD plants using both TRFLP analysis and amplicon sequencing (Illumina platform) of the 16S rRNA gene to determine the potential of TRFLP for a reliable capture of a useful ecological picture of the AD microbiome. A comparison was made based on (1) alpha-diversity, (2) beta-diversity, (3) community organization, (4) the impact of operational parameters on the community profile, and (5) co-occurrence network formation to estimate the potential of TRFLP analysis on different levels.

## Material and Methods

### Sample collection and storage

A total of 33 unique anaerobic digester content samples were collected from 25 full-scale agricultural and industrial AD plants. Samples from the same plant were given the same name, but a different number, which represents different sampling time points. Sample collection and storage was carried out as described by De Vrieze, *et al*.^42^. The pH was measured directly upon arrival in the lab. Samples for total ammonia nitrogen (NH_4_^+^ and NH_3_), conductivity, volatile solids and total solids measurement were stored at 4 °C. Samples for VFA content analysis and DNA extraction were stored at −20 °C until further analyses. Information concerning the digester capacity, biogas production rate, organic loading rate, sludge retention time, feedstock composition and temperature was obtained directly from the plant operators.

### Microbial community analysis

#### DNA extraction

The DNA extraction was carried out with the FastDNA^®^ SPIN Kit for Soil (MP Biomedicals, Solon, OH, USA), starting from 200 mg of digester content, in accordance with the instructions of the manufacturer. The quality of the DNA was validated with 1% agarose gel electrophoresis and PCR analysis to make sure that there were no PCR inhibiting components present in the DNA extracts. The control PCR amplification was performed according to the protocol of Boon, *et al*.^55^, using the bacterial primers P338F and P518R^56^ targeting the 16S rRNA gene. The PCR product quality, *i.e*., fragment length and the absence of non-desirable DNA fragments, confirmed that there was no PCR inhibition.

#### Amplicon sequencing and data processing

The DNA extracts were subjected to amplicon sequencing of the variable region 4 (V4) using the primers 515 F (5′-GTGCCAGCMGCCGCGGTAA-3′) and 806 R (5′-GGACTACHVGGGTWTCTAAT-3′) on the Illumina HiSeq platform with the protocol of Caporaso, *et al*.^57^. The PCR reaction was carried out in triplicate for each sample, and the PCR mixture and conditions, as well as PCR product purification and quantification were carried out as described by De Vrieze, *et al*.^42^.

The amplicon sequences were trimmed and filtered with Sickle v1.200^58^, using a sliding window approach. Reads with an average quality score below 20 were removed. Error correction was performed with the BayesHammer error correction tool^59^, coupled with the Spades v2.5.0 assembler. The paired-end reads were assembled with PANDAseq v2.4^60^ with a minimum overlap of 20. This three-step approach was used to obtain up to 90% error reduction^61^. Taxonomic unit construction was carried out with the UPARSE (v7.0.1001) pipeline^62^. The reads were dereplicated, and sorted by decreasing abundance. The absolute singletons were discarded, followed by simultaneous chimera filtering and OTU (operational taxonomic unit) clustering, based on 97% similarity. An additional reference-based chimera filtering step was included, using the ‘gold’ database (http://drive5.com/uchime/gold.fa), derived from the ChimeraSlayer reference database in the Broad Microbiome Utilities (http://microbiomeutil.sourceforge.net). The standalone RDP Classifier v2.6^63^ was used to classify representative sequences from the OTUs against the Ribosomal Database Project (RDP) with a confidence threshold of 80%. A table with the abundance of the different OTUs and their taxonomic assignments, and containing each sample was generated, containing re-sequenced samples as individual sample libraries. Samples were sent out for re-sequencing when sampling depth was insufficient (<10,000 reads).

#### TRFLP analysis

The TRFLP analyses were carried out following the protocols proposed by Rademacher, *et al*.^20^ and Klang, *et al*.^21^. Briefly, the bacterial and archaeal 16S rRNA genes were amplified (three replicates per crude DNA extract) using the primer pairs 27 F/926MRr (Bacteria) and Ar109f/Ar912r (Archaea), whereby the forward primers were fluorescently labelled with Cy5. After purification of the PCR products, 150–200 ng was digested with *Msp*I and *Hin*6I for the bacterial and with *Alu*I for the archaeal assay. The digestion fragments were separated using a GenomeLab™ GeXP Genetic Analysis System (AB SCIEX Germany GmbH, Darmstadt, Germany). The obtained data were pre-analysed using the GeXP analysis software (version 10.2), whereby only profiles were considered for further analyses whose internal standard had a standard deviation of 0.39 nucleotides (nt) or less^20^. A detailed analysis was performed using the software package BioNumerics 7.6 (Applied Maths, Kortrijk, Belgium), according to Klang, *et al*.^21^.

### Statistical analyses

The statistical analyses were carried out in R studio, version 3.3.1 (http://www.r-project.org)^64^, using the packages vegan^65^ and phyloseq^66^ for microbial community analysis. Prior to data processing, repeated measures analysis of variance (ANOVA, *aov* function) was used to confirm that relative abundance profiles of re-sequenced samples were not significantly different after which samples were collated as described by Connelly, *et al*.^67^. All samples were rescaled by taking the proportions of each OTU, multiplying it with the minimum sample size, and rounding to the nearest integer^68^. Only OTUs with a relative abundance ≥1% were considered both for the Illumina and TRFLP data to allow accurate and direct comparison between the two methods. Significant differences in the order-based Hill’s numbers^69^ reflecting richness (number of OTUs or TRFs, H_0_), the exponential of the Shannon diversity index (H_1_) and the Inverse Simpson index (H_2_), between the amplicon and TRFLP data were determined with ANOVA. Spearman’s Rank correlations between the Hill numbers obtained from the amplicon and TRFLP data were determined using the *ddply* function (plyr package). The non-metric multidimensional scaling (NMDS) plots were constructed based on the Bray-Curtis^70^, Chao^71^, Jaccard, Kulczynski^72^, and Mountford^73^ distance measures. Correlations between the amplicon and TRFLP distance measures were determined using the Spearman’s and Kendall Rank correlation. Permutational ANOVA (PERMANOVA) (9999 permutations) with Bonferroni correction was used to determine significant differences in community composition between different clusters, and to evaluate the influence of the operational parameters on the amplicon and TRFLP data profiles using the *adonis* function (vegan). A canonical correspondence analysis (CCA) model, using the *envfit* function (vegan) was used to evaluate the strength of the correlation of the operational parameters (Table S1). Operational parameters with a significant impact on the microbial community profile were determined through PERMANOVA analysis (9999 permutations), and visualised with canonical correspondence analysis plotting. The community organization (Co) was calculated based on the Lorenz distribution curves^45,74^, and the Pareto value was calculated as the total relative abundance of the 20% most abundant OTUs or TRFs^75,76^. Significant differences between the amplicon and TRFLP data and Spearman’s Rank correlations were determined, similar as for the Hill numbers. Co-occurrence networks were constructed, based on the Spearman’s rank correlation, with the igraph (http://igraph.org), sna^77^ and network^78^ packages, and the network statistics betweenness centrality and normalised degree were calculated^79^.

### Analytical techniques

Total solids (TS), volatile solids (VS) and total ammonia nitrogen (NH_4_^+^ and NH_3_) were determined via Standard Methods^80^. The total ammonia nitrogen, pH and temperature were used to calculate the free ammonia (NH_3_) values^81^. The different VFA concentrations were measured through gas chromatography, as described in SI (S1). Conductivity was measured with a C833 conductivity meter (Consort, Turnhout, Belgium), and pH was determined using a C532 pH meter (Consort, Turnhout, Belgium).

## Results

### Operational data of the full-scale anaerobic digesters

Samples were collected from the 25 different full-scale digesters, with a total volume between 1000–4000 m^3^, during a period of steady state feedstock supply and stable biogas production (<10% variation) of at least one year. The different digesters had a wide range of feedstock compositions, differences in sludge retention time (18–124 days), organic loading rate (1.5–13.8 kg COD m^−3^ d^−1^) and biogas production (1.1–7.5 m^3^ m^−3^ d^−1^) (Table S1). This corresponded with different pH (7.10–8.52), total ammonia nitrogen (130–6400 mg N L^−1^), free ammonia nitrogen (1.7–1460 mg N L^−1^), and conductivity (6.4–62.3 mS cm^−1^) ranges, and total VFA concentrations of up to 36.8 g COD L^−1^. Most samples (62%) were collected from mesophilic digesters (33–38 °C), while one sample was collected from a digester operating at 42 °C, and the other samples (35%) were collected from thermophilic digesters (50–55 °C).

### Microbial community profiling

Amplicon sequencing of the V4 region of the 16S rRNA gene yielded an average of 40,731 ± 16,647 reads from in total 4,027 different OTUs, with on average of 703 ± 287 OTUs per sample. No significant differences (*P* < 0.05) were observed between samples that were sequenced multiple times. Rarefaction curves indicate that saturation was not reached for all samples (Figure S1), but to allow comparison between the Illumina and TRFLP data, only OTUs with a relative abundance ≥1% in at least one sample were considered. Hence, this did not influence further data processing and interpretation. The 1% cut-off value resulted in total in only 241 different OTUs being retained for further analysis. Using a cut-off value of 1% relative abundance in at least one sample in the Illumina data resulted in an elimination of all archaeal OTUs.

The TRFLP community profiling yielded a total of 142 different bacterial and 12 different archaeal terminal restriction fragments (TRFs), with an average of 16 ± 1 and 4 ± 1 bacterial and archaeal TRFs per sample, respectively. Following the application of the 1% cut-off value, the total number of bacterial TRFs decreased to 120, while no archaeal TRFs were removed. All further analyses were carried out using the datasets on which the 1% cut-off was applied.

### Microbial community diversity and organization

**Figure 1:**
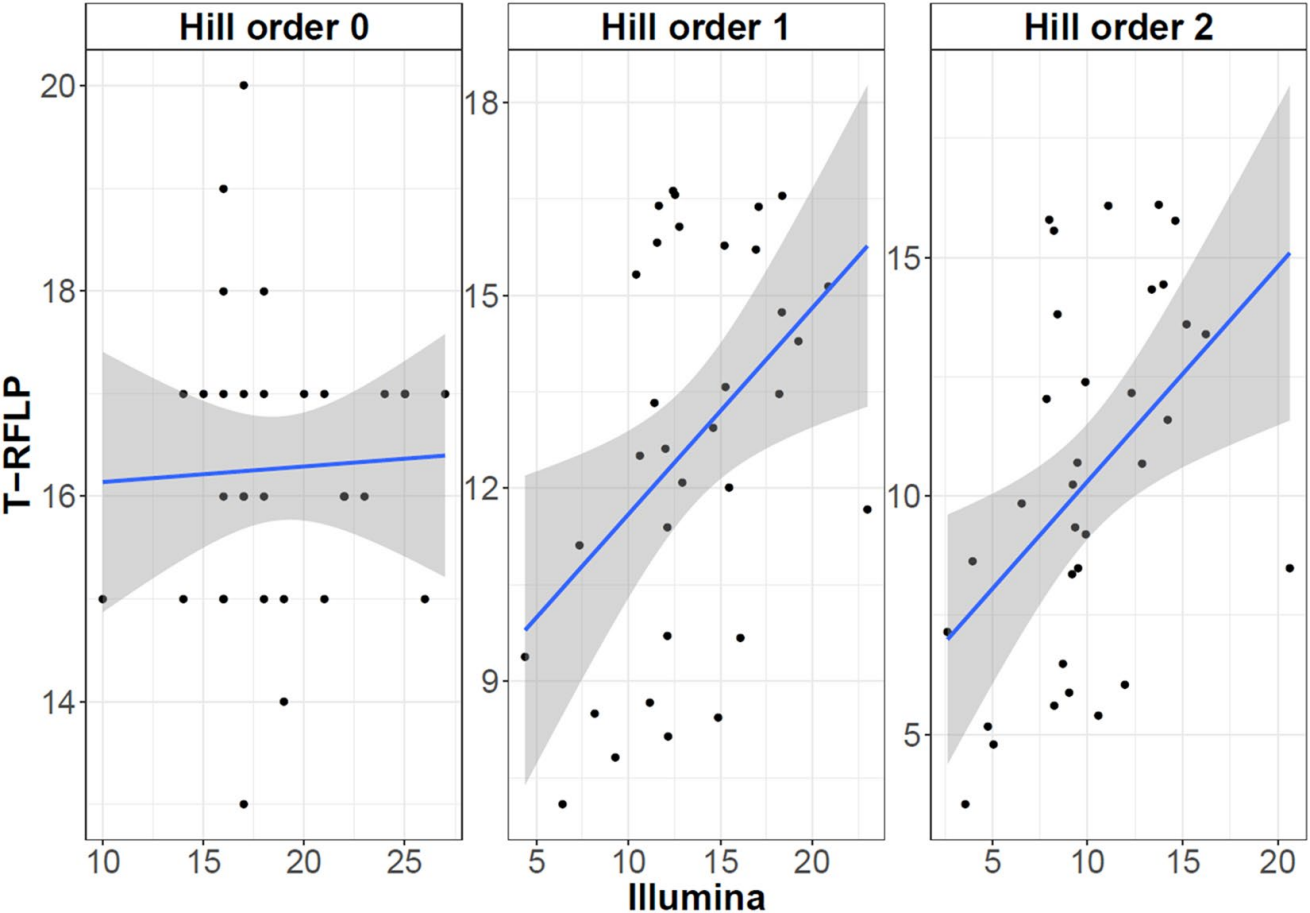
Spearman’s Rank correlation for the H_0_, H_1_ and H_2_ Hill numbers. The richness (H_0_), exponential of the Shannon diversity index (H_1_) and the Inverse Simpson index (H_2_) are included. Correlation between the Illumina and TRFLP data for H_0_ (*P* = 0.87) was not significant, yet, for H_1_ (*ρ* = 0.37 & *P* = 0.033) and H_2_ (*ρ* = 0.42 & *P* = 0.015) a significant positive correlation was observed. The grey zone represents the 95% confidence interval.

Spearman’s Rank correlation analysis of the microbial diversity parameters, determined via the Hill’s numbers, revealed that there was no significant correlation between the Illumina and bacterial TRFLP data for H_0_ (*P* = 0.87), but for H_1_ (*ρ* = 0.37 & *P* = 0.033) and H_2_ (*ρ* = 0.42 & *P* = 0.015) a significant positive correlation was observed (Fig. 1). A significantly higher richness (H_0_) value (*P* = 0.0007) was observed for the Illumina based analysis than for the bacterial TRFLP data (Figure S2). No significant difference was, however, observed between the Illumina and bacterial TRFLP data for the H_1_ (*P* = 0.39) and H_2_ (*P* = 0.78) Hill’s numbers. The archaeal TRFLP data showed significantly (*P* < 0.0001) lower H_0_, H_1_ and H_2_ values compared with the bacterial TRFLP, which was a consequence of the fact that, on average, only 4 ± 1 TRFs were observed per sample for the archaeal TRFLP (Figure S2).

**Figure 2:**
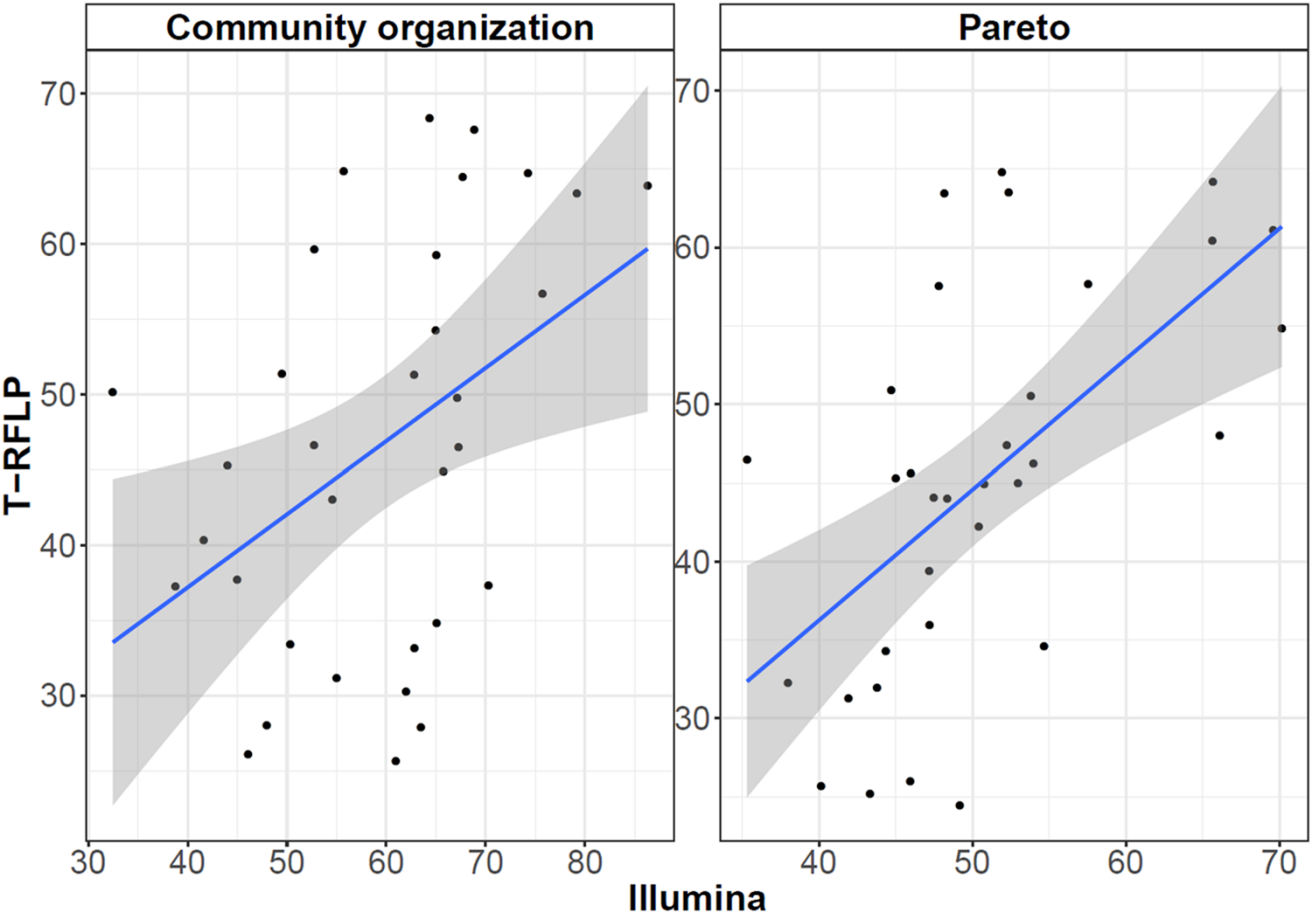
Spearman’s Rank correlation for the community organisation (Co) and Pareto values. The Co was calculated based on the Lorenz distribution curves^45,74^, and the Pareto value was calculated as the total relative abundance of the 20% most abundant OTUs or TRFs^75,76^. A significant positive correlation between the Illumina and TRFLP data was observed for Co (*ρ* = 0.45 & *P* = 0.009) and Pareto (*ρ* = 0.56 & *P* = 0.0009). The grey zone represents the 95% confidence interval.

A significant positive correlation could be observed between the Illumina and bacterial TRFLP data, both for the community organisation (Co) (*ρ* = 0.45 & *P* = 0.009) and Pareto (*ρ* = 0.56 & *P* = 0.0009) values (Fig. 2). Community organisation analysis showed a significantly higher (*P* = 0.0002) average Co value of 59.4 ± 12.2 for the Illumina than for the bacterial TRFLP data (46.6 ± 13.6), indicating a more evenly distributed community based on the bacterial TRFLP data (Fig. 2). The community organisation based on the archaeal TRFLP data was significantly higher (*P* < 0.0001) than based on the bacterial TRFLP data (Figure S3). A similar significant difference (*P* = 0.038) was observed between the Illumina and bacterial TRFLP data for the Pareto values, which confirms the apparent higher evenness based on the bacterial TRFLP data. The average Pareto value was significantly higher (*P* < 0.0001) for that archaeal TRFLP than for the bacterial TRFLP data, but this was a consequence of the skewed archaeal TRFLP Pareto profile, related to the low TRFs number.

### Beta-diversity analysis

**Figure 3:**
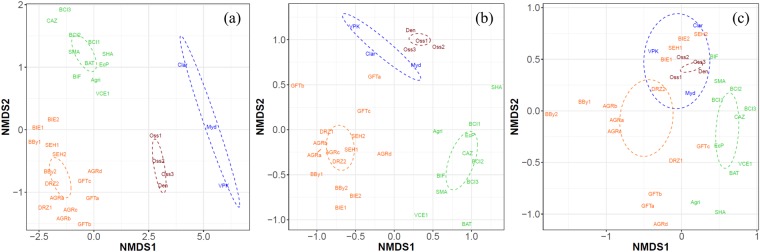
Non-metric distance scaling (NMDS) analysis of the Bray-Curtis dissimilarity distance indices of the (**a**) Illumina (stress = 0.09), bacterial TRFLP (stress = 0.24), and (**c**) archaeal TRFLP (stress = 0.17) at OTU/TRF level. The four different clusters Cluster 1 (red), Cluster 2 (blue), Cluster 3 (green), and Cluster 4 (orange) are distinguished, and the ellipses represent the 95% value of the standard error of the average value for each cluster.

For both Illumina and bacterial TRFLP data, the NMDS analysis of the Bray-Curtis dissimilarity matrix revealed the presence of four apparent clusters (Fig. 3). Cluster 1 contained samples from sludge digesters (Den, Oss1, Oss2, and Oss3), characterized by the lowest values of the operational parameters (Table S1), while cluster 2 contained samples from upflow anaerobic sludge bed reactors treating potato or paper mill wastewater (Clar, Myd and VPK). Cluster 3 contained mainly samples from mesophilic digesters treating bio- and agricultural wastes (BCI, SMA, SHA, BAT, BIF, Agri, CAZ, EcP, VCE), and cluster 4 samples from thermophilic digesters (GFT, AGR, DRZ, BBy, BIE), although the mesophilic SHE digester was also included in cluster 4. A significant correlation could be observed between the Bray-Curtis dissimilarity matrix for the Spearman’s (ρ = 0.17 & *P* = 0.0001) and Kendall (τ = 0.12 & *P* = 0.0002). The PERMANOVA analysis confirmed that clusters 2, 3 & 4 were significantly separated (*P* < 0.05), based on the Illumina and bacterial TRFLP data, but that cluster 1 was not (*P* = 0.17 for Illumina and *P* = 0.17 for bacterial TRFLP data). The same pattern of clustering was detected for data obtained using both the Illumina and bacterial TRFLP methods. Significant differences between the clusters for the archaeal TRFLP data could be observed only between cluster 1 and 3 (*P* = 0.006), cluster 2 and 3 (*P* = 0.040) and cluster 3 and 4 (*P* = 0.0018). A similar observation was made through the NMDS analysis of the Chao, Jaccard, Kulczynski, and Mountford distance measures (Figure S4).

**Figure 4:**
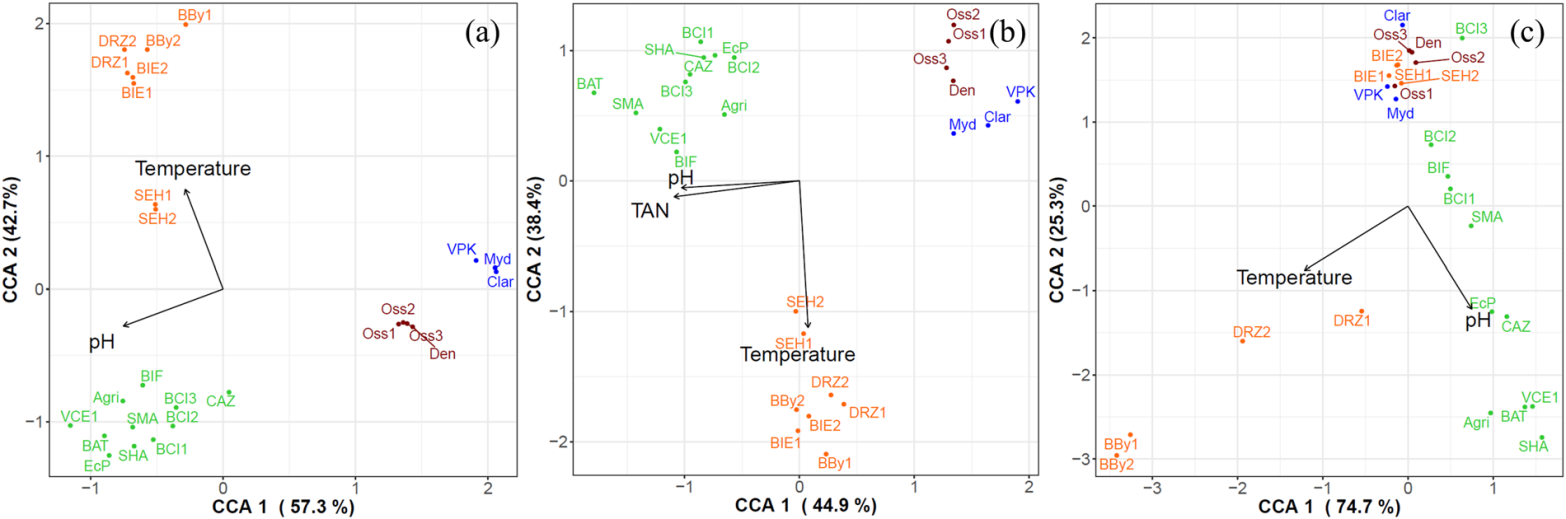
Canonical correspondence analysis of the (**a**) Illumina, (**b**) bacterial TRFLP and (**c**) archaeal TRFLP profile of each sample at OTU/TRF level. The PERMANOVA analysis (9999 permutations) identified the relationship between the diversity and operational parameters on community composition, and significant (*P* < 0.05) correlations are presented by the arrows. The four different clusters Cluster 1 (red), Cluster 2 (blue), Cluster 3 (green), and Cluster 4 (orange) are distinguished. TAN = total ammonia nitrogen.

Canonical correspondence analysis demonstrated that the pH and temperature had a significant impact on the Illumina (*P*_pH_ = 0.0001, *P*_temp_ = 0.0001), the bacterial TRFLP (*P*_pH_ = 0.001, *P*_temp_ = 0.002), as well as the archaeal TRFLP (*P*_pH_ = 0.002, *P*_temp_ = 0.002) profiles (Fig. 4, Table S2). The total ammonia concentration, however, had a significant impact for the bacterial TRFLP data (*P* = 0.007), but not for the Illumina (*P* = 0.064) and archaeal TRFLP data (*P* = 0.11). None of the other operational parameters had a significant impact on any of the three community profiles.

### Co-occurrence network analysis

**Figure 5:**
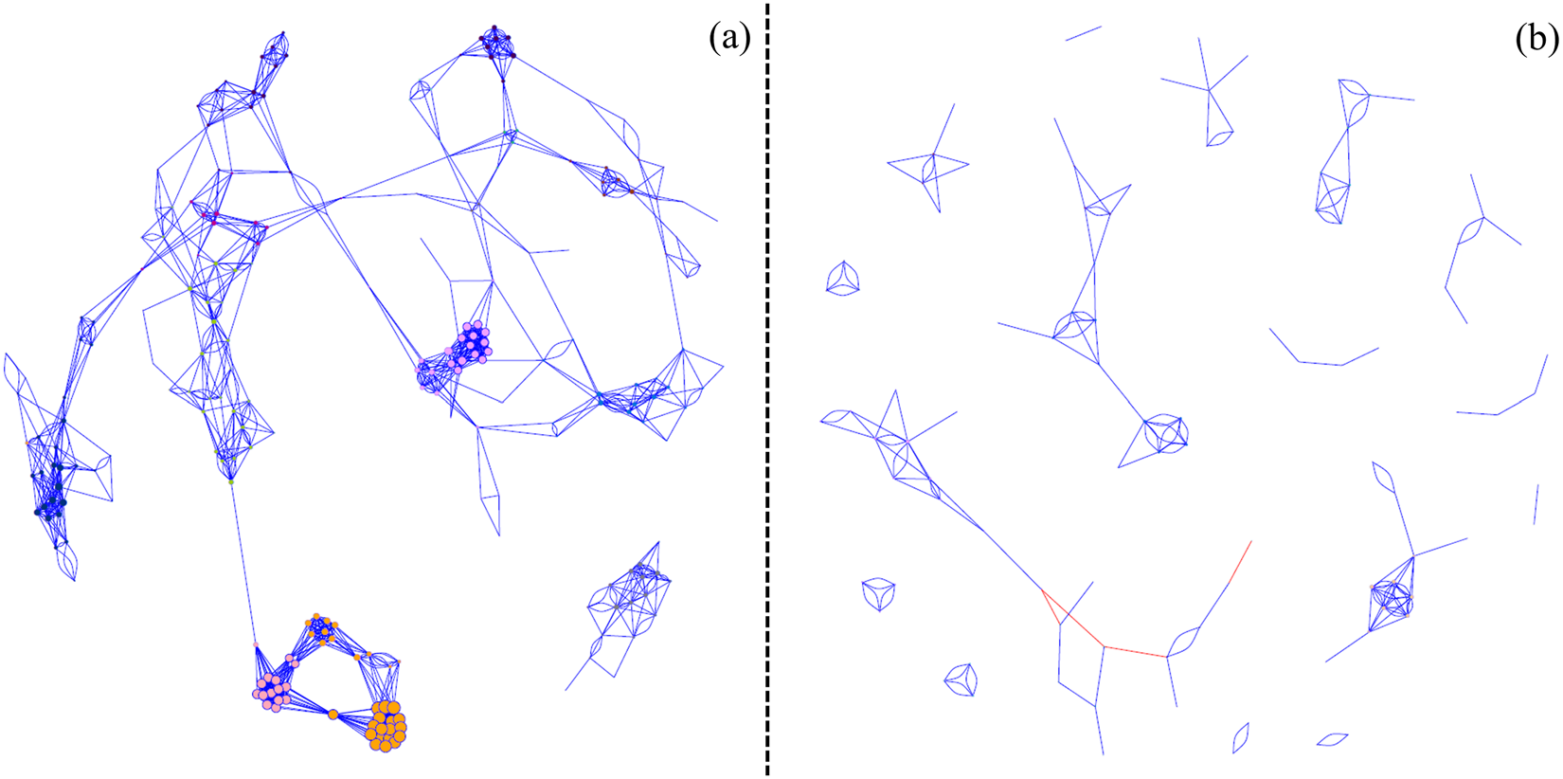
Network of co-occurring (**a**) OTUs of the Illumina and (**b**) TRFs of the bacterial data, based on the Spearman correlation analysis. A connection stands for a strong positive (blue) or negative (red) significant (Spearman’s *ρ* > 0.5, *P* < 0.001) correlation. The size of each node is proportional to the number of connections (Normalised degree).

Co-occurrence networks were constructed for the Illumina, bacterial TRFLP and archaeal TRFLP profiles to determine to which extent inter-taxa correlations and related network statistics are reflected in a similar manner. No significant (*P* < 0.001) correlations were observed for the archaeal TRFs probably due to the paucity of the data, thus, no additional analyses were carried out. A clear inter-taxa correlation could be observed in the co-occurrence network based on the Illumina data, while this was not the case for the bacterial TRFLP data, which resulted in a much more fragmented profile (Fig. 5). In total 1,494 significant correlations (*P* < 0.001) existed between the 241 OTUs recorded within the Illumina dataset, representing 5.2% of total potential correlations. The 120 bacterial TRFs contained 245 significant correlations, which corresponded with 3.4% of total potential correlations. Only 4 OTUs (1.7%) had no significant correlations, while 20 TRFs (16.7%) had no significant correlations.

**Figure 6:**
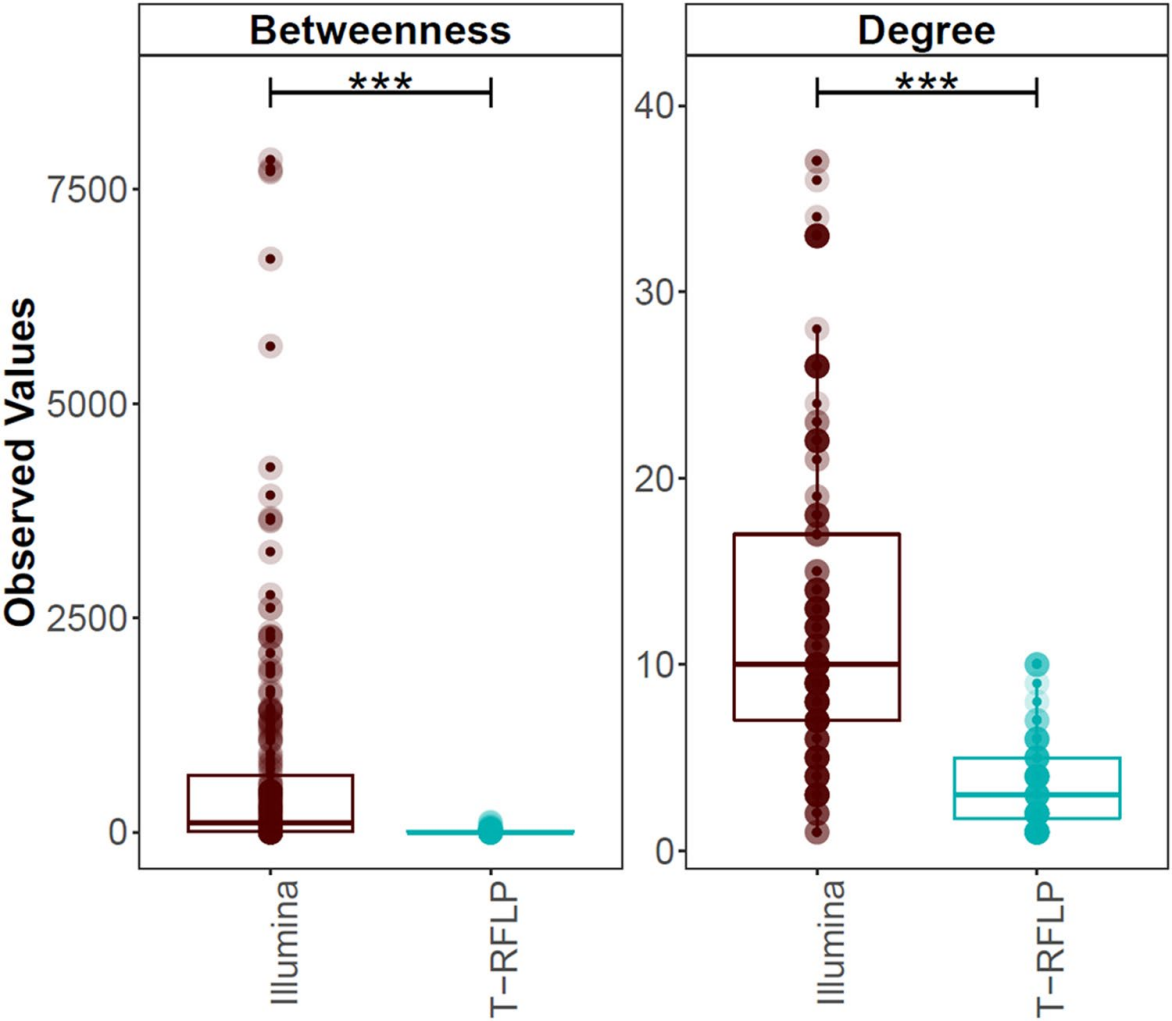
Betweenness centrality and normalised degree, calculated from the co-occurrence network correlations for the Illumina and bacterial TRFLP data. ***P < 0.001.

Betweenness centrality, which reflects the importance of an OTU or TRF as connector in the network^79^,was significantly higher (*P* < 0.0001) for the Illumina profile, compared with the bacterial TRFLP profile (Fig. 6). Out of 237 OTUs with at least one significant correlation, 86.9% had a betweenness score >0, while out of 100 TRFs with at least one significant correlation, only 47.0% had a betweenness score >0. The normalised degree, which reflects the proportion of other OTUs/TRFs a certain OTU/TRF interacts with^79^, showed a similar significantly higher (*P* < 0.0001) value for the Illumina network compared with the bacterial TRFLP network.

## Discussion

A comparison of the microbiomes in 25 full-scale AD plants was carried out to determine whether TRFLP profiling can be used as a simplified *proxy* of 16S rRNA gene amplicon (Illumina platform) sequencing. While the Illumina and bacterial TRFLP data showed a high degree of similarity at higher-order α-diversity levels, a significant difference in richness was observed. The archaeal TRFLP diversity was significantly lower than the bacterial TRFLP diversity, related to the structured bacteria-archaea succession in stable anaerobic digesters^82,83^. The β-diversity analysis revealed a similar pattern for the Illumina, bacterial TRFLP and archaeal TRFLP data. This was, however, not reflected in a similar co-occurrence pattern, related to significant differences in betweenness centrality, normalised degree, community organisation and Pareto distribution between the Illumina and bacterial TRFLP data.

### The (non)sense of richness in anaerobic digestion

Microbial community richness has been one of the most commonly used parameters to estimate taxonomic diversity and the related metabolic potential, carrying physiological/ecological capacity and overall stability of a highly diverse and complex microbial community in different natural or engineered ecosystems^45,84–88^. Although a high richness does not necessary or directly reflect a high metabolic potential, it has been suggested to increase the resilience and/or resistance of microbial communities^89^, and both parameters contribute to process stability and efficiency in AD^90–92^.

Microbial community richness, however, highly depends on the method used to determine it, as observed not only in our study, but also in previously published studies^93,94^. While based on the Illumina data an average richness of 19 ± 4 OTUs was observed per sample, this was 16 ± 1 TRFs for the bacterial TRFLP data, and only 4 ± 1 TRFs for the archaeal TRFLP data. The low archaeal richness can be explained by the fact that the methanogenic archaea only contribute to the final stage of the AD process, using a narrow substrate spectra, hence, representing a low (relative) abundance, often <5%^24^. This is also reflected in the fact that using a cut-off value of 1% relative abundance in at least one sample in the Illumina data resulted in an elimination of all archaeal OTUs. Inherently, this coincides with a low archaeal richness.

The significant difference in richness between the Illumina and bacterial TRFLP data is more striking, as both were normalised via the common-scale method^68^ and subjected to a 1% cut-off value. This raises the question what can be considered as the relevant richness, because “total richness” coverage in AD, as determined *via* rarefaction curves, was not obtained in this study, and is rarely met through 16S rRNA gene amplicon sequencing^31,42^. The importance of the rare or uncommon taxa remains open for debate^95,96^, but it is clear that deep sequencing is needed to determine the absolute richness^93^. Even then, method-inherent choices, such as the clustering method and threshold^97^, will strongly influence absolute richness values. Hence, richness as an indicator of microbial stability is too much dependent on the method used to measure it, and may not be useful to define the diverse microbial ecosystem in AD. It raises the question what depth of resolution is useful to identify parameters for process control based on microbial diversity.

### Beta-diversity analysis: fingerprinting technique independent

The method-dependence of richness also affected the β-diversity metrics community organisation (Co), Pareto distribution, and co-occurrence networking and related statistics. Their strong dependency on the richness value, especially at low richness values^79,98,99^ explains the significant differences between the Illumina and bacterial TRFLP data, and warrants careful interpretation. In contrast, no significant difference was recorded between the Illumina and bacterial TRFLP regarding the exponential of the Shannon diversity index (H_1_) and the Inverse Simpson index (H_2_), whereby the Spearman’s Rank analysis revealed a significant positive correlation between both data sets. The high similarity of the β-diversity profiles, based on the Illumina, bacterial TRFLP and even archaeal TRFLP data is apparent. This similarity seems to be distance measure independent, as the Bray-Curtis, Chao, Jaccard, Kulczynski, and Mountford distance measures revealed a similar clustering pattern.

The robustness of β-diversity analysis in AD is supported by the fact that canonical correspondence analysis determined pH and temperature as the two operational parameters with a significant impact on the microbiome in the studied full-scale anaerobic digesters, highlighted *via* the Illumina, bacterial TRFLP and archaeal TRFLP community profiles. The strong impact of pH, mainly related to free ammonia toxicity^19,100^, and temperature on microbial community composition has been observed in several other studies^32,42,101^. Although total ammonia nitrogen had a significant impact only on the bacterial TRFLP community profile, its insignificance towards the Illumina community profile needs to be considered with care, given the low P-value of 0.064, which only marginally exceeded the threshold value of 0.05 and high R^2^ value of 0.856, based on the CCA model. The robustness of β-diversity, hence, also depends on the direct interpretation of P-values, rather than considering a “significant on/off effect”.

The reliability and reproducibility of β-diversity analysis through different fingerprinting methods has been observed in other ecosystems, and can be applied, if allowed by the method, on different phylogenetic levels^102^. Although the bacterial TRFLP data in most cases did not allow comparison at deeper phylogenetic levels (*e.g*., family or genus), they do provide an unambiguous bacterial fingerprint, independent of the taxonomy assignment. Hence, TRFLP can be used as a robust fingerprinting method for β-diversity analysis in AD, as reflected in its similarity with high-throughput amplicon sequencing based β-diversity profiles.

### Towards practical applications: microbial community profiling in AD

The robustness of TRFLP analysis for microbial community β-diversity profiling in AD provides a suitable framework for microbial community screening to describe the dominant ecological diversity, related to the 1% cut-off, reflecting the interactions of the dominant microorganisms among each other and with their environment. While high-throughput 16S rRNA gene amplicon sequencing techniques provide a more in-depth microbial community profile, the data evaluation time constraints seriously hamper application on the full-scale level regarding the high flexibility of the microbiomes against changing environmental factors, and, hence, the need for long-term temporal observation/process control. As functional stability in AD does not necessarily reside on the presence of specific species, rather than an overall high functional redundancy^92,103^, fingerprinting techniques can be used, alternative to molecular techniques that enable identification of the microbial community members, for AD process stability screening. However, this does not exclude the potential importance of certain “abundant core” OTUs in AD^104–106^. The accurate definition of boundary conditions with respect to microbial community variation over time and/or in response to disturbances is of crucial importance to guarantee the application of microbial community profiling in AD. As the microbial communities in lab- and full-scale plants displays continuous natural dynamics in its taxonomic, functional and ecological diversity over time^15,21,83,107,108^, it is necessary to distinguish natural variation from disturbance-related unwanted community shifts that lead to process deterioration. The unique community structure in each AD plant, even considering temporal dynamics^21,91,107^, related to the feedstock characteristics and operational conditions^32,109^, is a first important consideration. Second, full-scale plants cluster according to feedstock (Cluster 1), digester type (Cluster 2) and operational conditions, *e.g*., pH, temperature and free/total ammonia concentration, as also observed in other studies^32,42,104^. When combining the uniqueness of the AD plant, considering its temporal variation at constant and stable conditions, with its expected place in the full-scale plant clustering or AD-typing, β-diversity analysis based on the TRFLP profile can be used for swift microbial community monitoring of full-scale AD plants. Deviations from the natural temporal dynamics during stable conditions would require a corrective action through operational parameter adjustment by applying balanced microbial diversity management strategies, considering the living demands of the occurring microbiome, or alternative biological strategies, such as targeted bioaugmentation. At transient conditions, *e.g*., related to changes in feedstock composition or deliberate changes in the operational parameters, a shift of the microbial community outside the basic temporal variation, but within its functional redundancy can be expected. In that case, a new temporal dynamics window should be identified, based on (1) the stabilisation of operational parameters, (2) full-scale digesters with similar feedstocks and/or operational parameters, and (3) its expected place in the clustering or AD-typing.

## Conclusions

The comparison of the Illumina amplicon, bacterial TRFLP and archaeal TRFLP profiles of 25 full-scale AD plants revealed high degree of similarity in the β-diversity profiles. This suggests that TRFLP may be easier and cheaper, although this strongly depends on the available facilities, constantly evolving techniques and their related costs, alternative to amplicon sequencing for monitoring changes of the overall structure of the microbial communities in full-scale AD to maintain a stable and efficient biogas production process. Even today, in the era of next generation sequencing, the reliability of TRFLP is not diminishing, particularly for the study of microbial community dynamics in relation to the effects of changing environmental, especially process inhibiting, factors on the taxonomic, the functional, as well as the ecological diversity of AD microbiomes. Briefly, TRFLP is not at all obsolete, and will remain of high value in the field of microbial ecology.

## Electronic supplementary material

Dataset 1

Dataset 2

Dataset 3

General Supplementary Information

## Data Availability

The raw fastq files that were used to create the OTU table of the 16S rRNA gene amplicon sequencing data (Illumina HiSeq platform) have been deposited in the National Center for Biotechnology Information (NCBI) database (Accession number SRP132836). The resulting OTU table has been included in the Supplementary Information (Supplementary Table S3), as well as the tables containing the bacterial and archaeal raw TRFs (Supplementary Tables S4 AND S5). All other data generated or analysed during this study are included in this article (and its Supplementary Information files).
