# Supplementary material for: Terminal restriction fragment length polymorphism is an “old school” reliable technique for swift microbial community screening in anaerobic digestion
Source: Sci Rep. 2018 Nov 14;8:16818. doi: 10.1038/s41598-018-34921-7 (PMC6235954; doi:10.1038/s41598-018-34921-7)
Supplement: Supplementary file 4 — General Supplementary Information [file 41598_2018_34921_MOESM4_ESM.pdf]

Supplementary Information

Journal: Scientific Reports

Title: Terminal restriction fragment length polymorphism as an “old school” but reliable technique for swift microbial community screening in anaerobic digestion

Jo De Vrieze[1,*], Umer Z. Ijaz[2], Aaron M. Saunders[3], Susanne Theuerl[4]

[1] Center for Microbial Ecology and Technology (CMET), Ghent University, Coupure Links 653, B-9000 Gent, Belgium

[2] Infrastructure and Environment Research Division, School of Engineering, University of Glasgow, UK

[3] Department of Biotechnology, Chemistry and Environmental Engineering, Aalborg University, Sohngardsholmsvej 49, 9000 Aalborg, Denmark

[4] Leibniz Institute for Agricultural Engineering and Bioeconomy e.V. (ATB), Department Bioengineering, Max-Eyth-Allee 100, D-14469 Potsdam, Germany

* Correspondence to: Jo De Vrieze, Ghent University; Faculty of Bioscience Engineering; Center for Microbial Ecology and Technology (CMET); Coupure Links 653; B-9000 Gent, Belgium; phone: +32 (0)9 264 59 76; fax: +32 (0)9 264 62 48; E-mail: Jo.DeVrieze@UGent.be; Webpage: www.cmet.ugent.be.

Contents

S1. Volatile fatty acid analysis 3

S2. Operational data 1

S3. Rarefaction curves 1

S4. Microbial community diversity 2

S5. Microbial community organisation 3

S6. Non-metric multidimensional scaling analysis 4

S7. Canonical correspondence analysis model results 6

References 7

S1. Volatile fatty acid analysis

The different VFA concentrations (C2-C8) were measured through gas chromatography (GC-2014, Shimadzu®, The Netherlands) with a DB-FFAP 123-3232 column (30 m × 0.32 mm × 0.25 µm; Agilent, Belgium) and a flame ionization detector. Sulphuric acid and sodium chloride were used to condition the digestate samples (2 mL), and 2-methyl hexanoic acid was used as internal standard for diethyl ether extraction efficiency quantification. The extracts (1 µL) were injected at 200 ºC with a split ratio of 60 and a purge flow of 3 mL min^{-1}. The oven temperature was increased by 6 ºC min^{-1} from 110 ºC to 165 ºC, where it was maintained for 2 min. The flame ionization detector had a temperature of 220 ºC. Nitrogen gas was used as carrier gas at a flow rate of 2.49 mL min^{-1}. The detection limit was 30 mg L^{-1} for acetate, 10 mg L^{-1} for propionate and 2 mg L^{-1} for the other VFA (C4-C8).

S2. Operational data

Table S1 Overview of the operational parameters of the different full-scale anaerobic digestion plants. Samples with the same name and a different number originate from the same anaerobic digestion plant at a different time point. The operational parameters were measured at the same time point the samples were taken. OLR = organic loading rate, SRT = sludge retention time, TS = total solids, VS = volatile solids, VFA = volatile fatty acids, TAN = total ammonia, FA = free ammonia, CSTR = continuously stirred tank reactor, UASB = upflow anaerobic sludge blanket, OBW = organic biological waste, OFMSW = organic fraction municipal solid waste, MSW = municipal solid waste, n.a. = data not available.

**Name**	**Type**	**Capacity**	**Feedstock**	**OLR**	**SRT**	**Temperature**	**TS**	**VS**	**pH**	**Conductivity**	**Total VFA**	**TAN**	**FA**	**Biogas**
		m^3		kg_{COD} m^{-3} d^{-1}	d	°C	g L^{-1}	g L^{-1}	[-]	mS cm^{-1}	g_{COD} L^{-1}	g L^{-1}	mg L^{-1}	m^3 m^{-3} d^{-1}
GFTa	CSTR	3150	OFMSW	13.8	22	50	154	131	8.5	n.a.	0.1	1.7	816	6.25
GFTb	CSTR	3150	OFMSW	8.8	25	52	109	87	8.3	n.a.	3.3	1.5	579	3.25
GFTc	CSTR	3450	OFMSW	6.0	38	50	309	146	8.2	n.a.	0.0	2.7	870	3.25
AGRa	CSTR	1200	Maize, manure	13.8	124	55	319	138	n.a.	n.a.	2.4	1.4	n.a.	11
AGRb	CSTR	1200	Maize, manure	13.8	33	55	389	171	n.a.	n.a.	0.0	1.4	n.a.	11
AGRc	CSTR	1200	Maize, manure	13.8	23	55	418	119	n.a.	n.a.	n.a.	1.4	n.a.	11
AGRd	CSTR	1200	Maize, manure	6.3	23	42	349	154	n.a.	n.a.	5.7	2.1	n.a.	2.5
DRZ1	CSTR	1000	Slaughterhouse waste	11.0	20	54	24	17	8.0	30.5	11.1	2.4	696	7.5
DRZ2	CSTR	1000	Slaughterhouse waste	11.0	20	54	51	35	8.0	25.0	11.1	2.4	696	7.5
SEH1	CSTR	1200	Maize, manure	4.0	40	34	58	33	8.0	40.1	4.7	3.5	338	1.1
SEH2	CSTR	1200	Maize, manure	4.0	40	34	59	34	8.1	39.5	4.5	3.5	380	1.1

BBy1	CSTR	3600	Manure, OBW, energy crops, slaughterhouse waste	4.5	40	54	42	28	7.5	32.3	6.6	3.3	364	1.5
BBy2	CSTR	3600	Manure, OBW, energy crops, slaughterhouse waste	5.6	40	54	45	29	7.5	32.5	7.6	3.2	353	2
BIE1	CSTR	2000	Maize, manure	4.0	30	54	109	67	7.9	38.5	8.1	3.1	743	7.4
BIE2	CSTR	2000	Maize, manure	4.0	30	54	109	68	7.9	38.2	7.9	3.3	701	7.4
BCI1	CSTR	1500	OBW	3.0	80	34	77	16	8.0	31.7	5.6	4.0	402	2.8
BCI2	CSTR	1500	OBW	3.0	80	34	44	24	8.0	31.7	22.6	2.7	268	2.8
BCI3	CSTR	1500	OBW	3.0	80	34	37	23	8.0	31.7	0.8	4.2	417	2.8
Oss1	CSTR	4000	Wastewater sludge	3.0	18	34	44	21	7.4	7.6	0.0	1.1	25	2
Oss2	CSTR	4000	Wastewater sludge	3.0	18	34	46	24	7.5	7.3	0.0	1.0	30	2
Oss3	CSTR	4000	Wastewater sludge	3.0	18	34	43	23	7.4	6.4	0.0	1.0	26	2
EcP	CSTR	n.a.	n.a.	n.a.	n.a.	34	21	13	8.3	39.2	0.0	3.1	540	n.a.
BAT	CSTR	n.a.	Manure, OBW	n.a.	n.a.	34	95	55	8.4	61.9	1.1	4.6	890	n.a.
VPK	UASB	1210	Paper mill wastewater	5.6	n.a.	35	165	85	7.2	6.9	0.3	0.2	4	1.2
Clar	UASB	n.a.	Potato wastewater	n.a.	n.a.	34	54	43	7.1	8.7	0.0	0.1	2	n.a.
VCE1	CSTR	1500	Maize, manure	1.5	100	38	127	77	8.5	28.6	5.7	4.6	1460	5.6
Myd	UASB		Potato wastewater	n.a.	n.a.	34	30	20	7.1	8.0	0.3	0.8	12	n.a.
Agri	CSTR	1000	Maize, fats, fruit waste	n.a.	40	34	133	48	8.2	37.5	0.9	2.9	410	5.7
Den	CSTR	3255	Sludge, manure	3.0	20	33	36	18	7.4	7.7	0.0	0.5	11	2.7
SHA	CSTR	1500	OBW	2.5	60	34	124	79	8.1	31.5	0.2	3.9	459	6.4
BIF	CSTR	1250	Manure	2.5	60	34	103	68	8.1	42.1	0.7	5.0	530	4.1
CAZ	CSTR	3000	Maize, manure	5.0	40	34	74	50	7.8	25.3	36.8	5.0	287	5.7
SMA	CSTR	3200	Maize, manure	4.0	40	34	112	64	8.3	62.3	0.4	6.4	1020	2.1

S3. Rarefaction curves

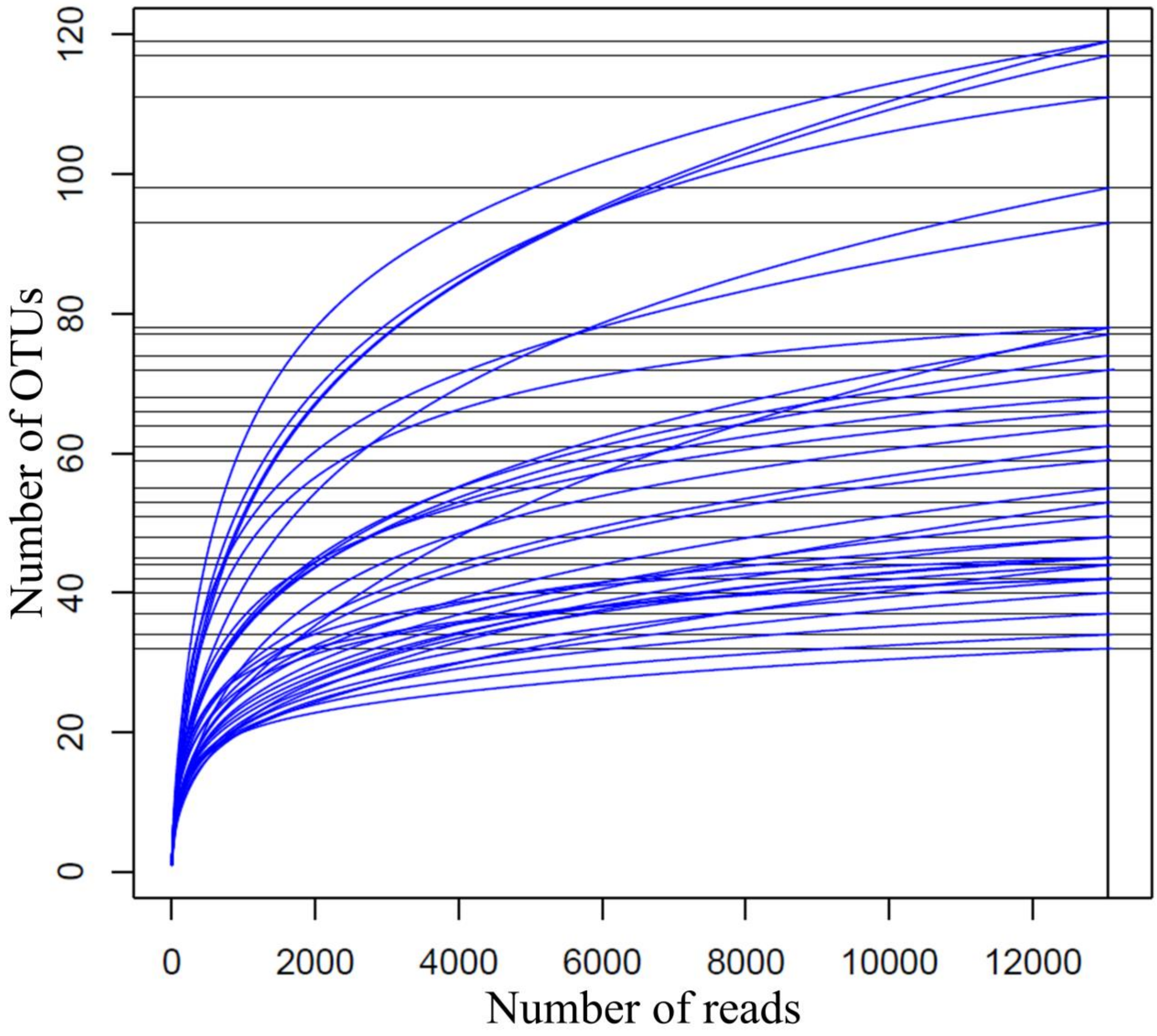

Figure S1 Rarefaction curves indicating the number of resolved OTUs against sampling depth of the different samples based on 16S rRNA gene amplicon sequencing (Illumina HiSeq platform). Data were normalised via the common-scale method [1], and only OTUs with a relative abundance ≥ 1 % in at least one sample were included.

S4. Microbial community diversity

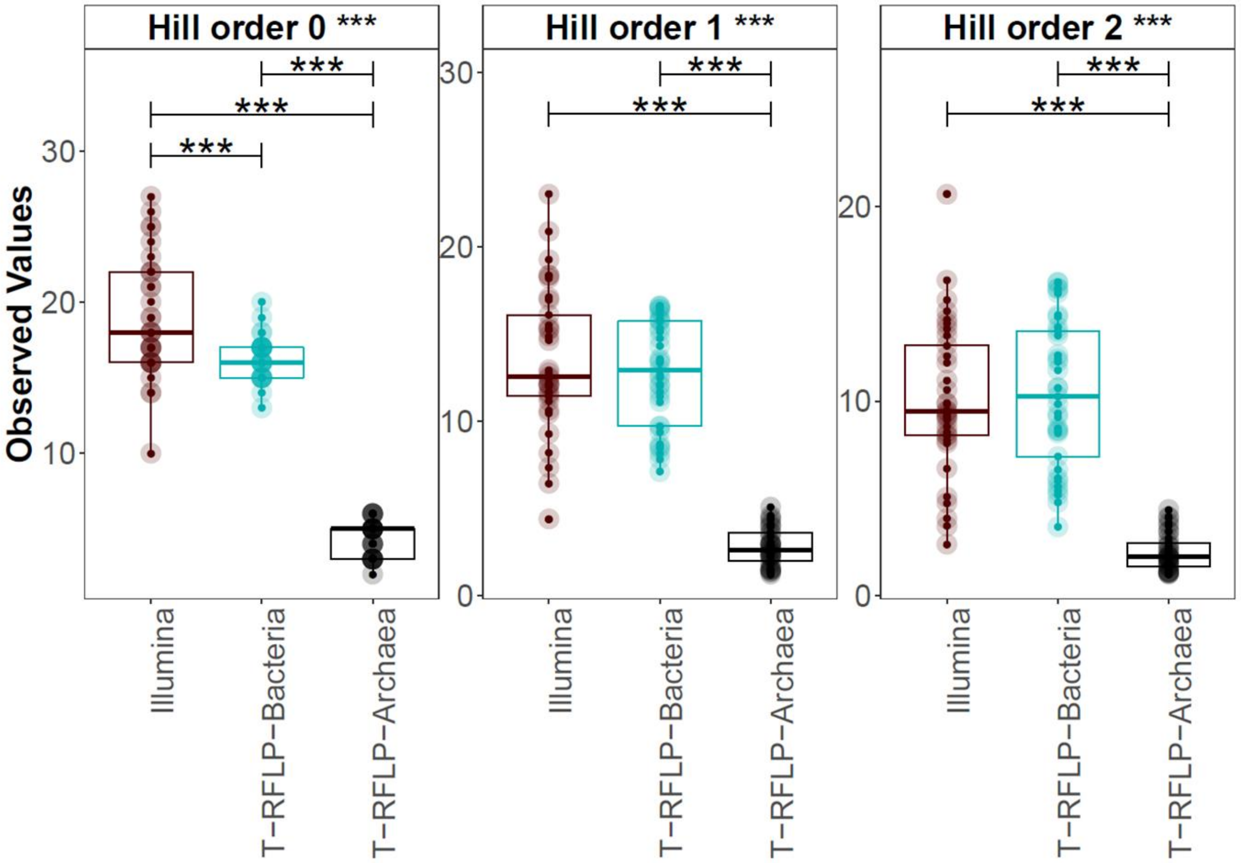

Figure S2 Hill's numbers of the Illumina, bacterial T-RFLP and archaeal T-RFLP data. The richness (H_0), exponential of the Shannon diversity index (H_1) and the Inverse Simpson index (H_2) are included. *** = $P < 0.001$.

S5. Microbial community organisation

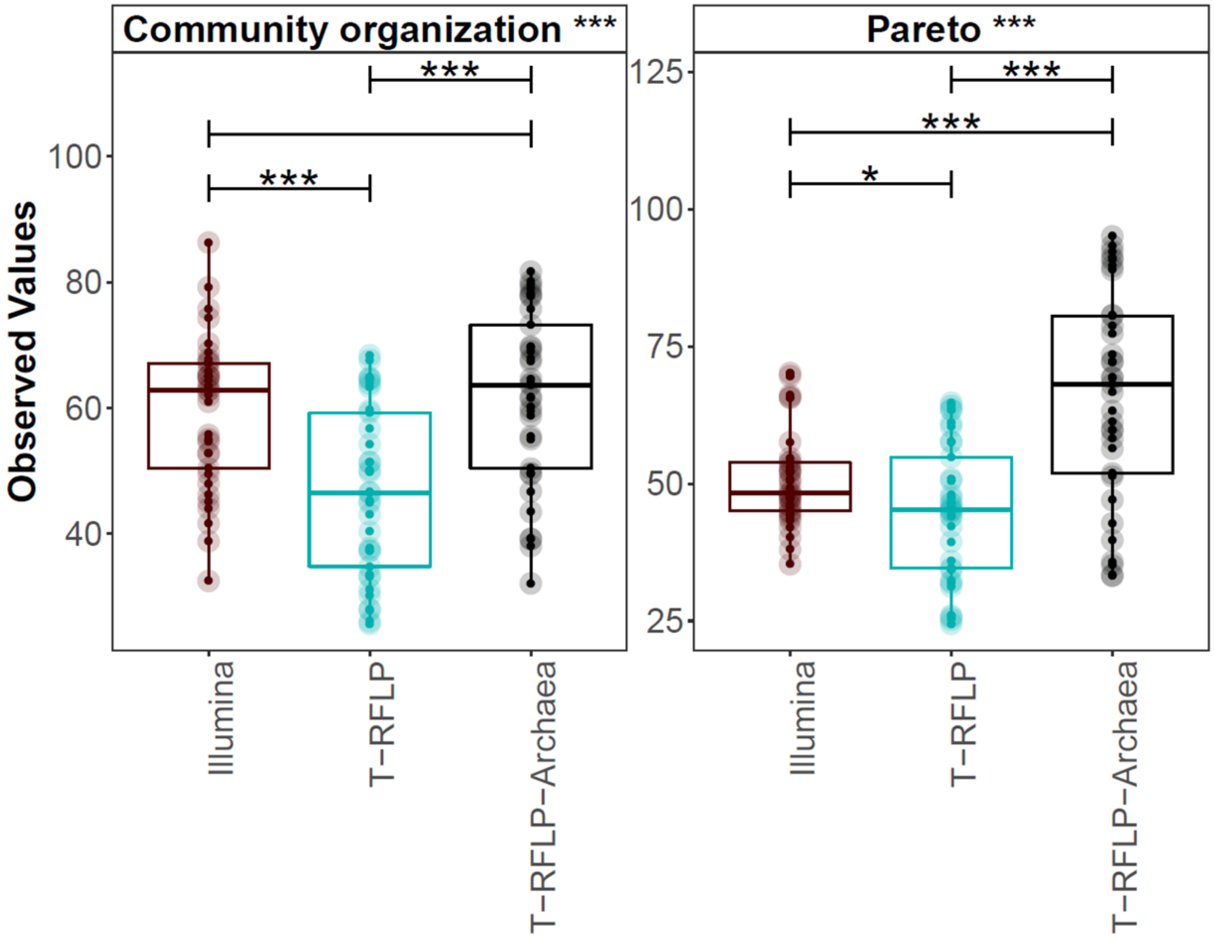

Figure S3 Community organisation (Co) and Pareto profile of the Illumina, bacterial T-RFLP and archaeal T-RFLP data. The Co was calculated based on the Lorenz distribution curves [2,3], and the Pareto value was calculated as the total relative abundance of the 20% most abundant OTUs or TRFs [4,5]. *=P < 0.05 & *** = P < 0.001.

S6. Non-metric multidimensional scaling analysis

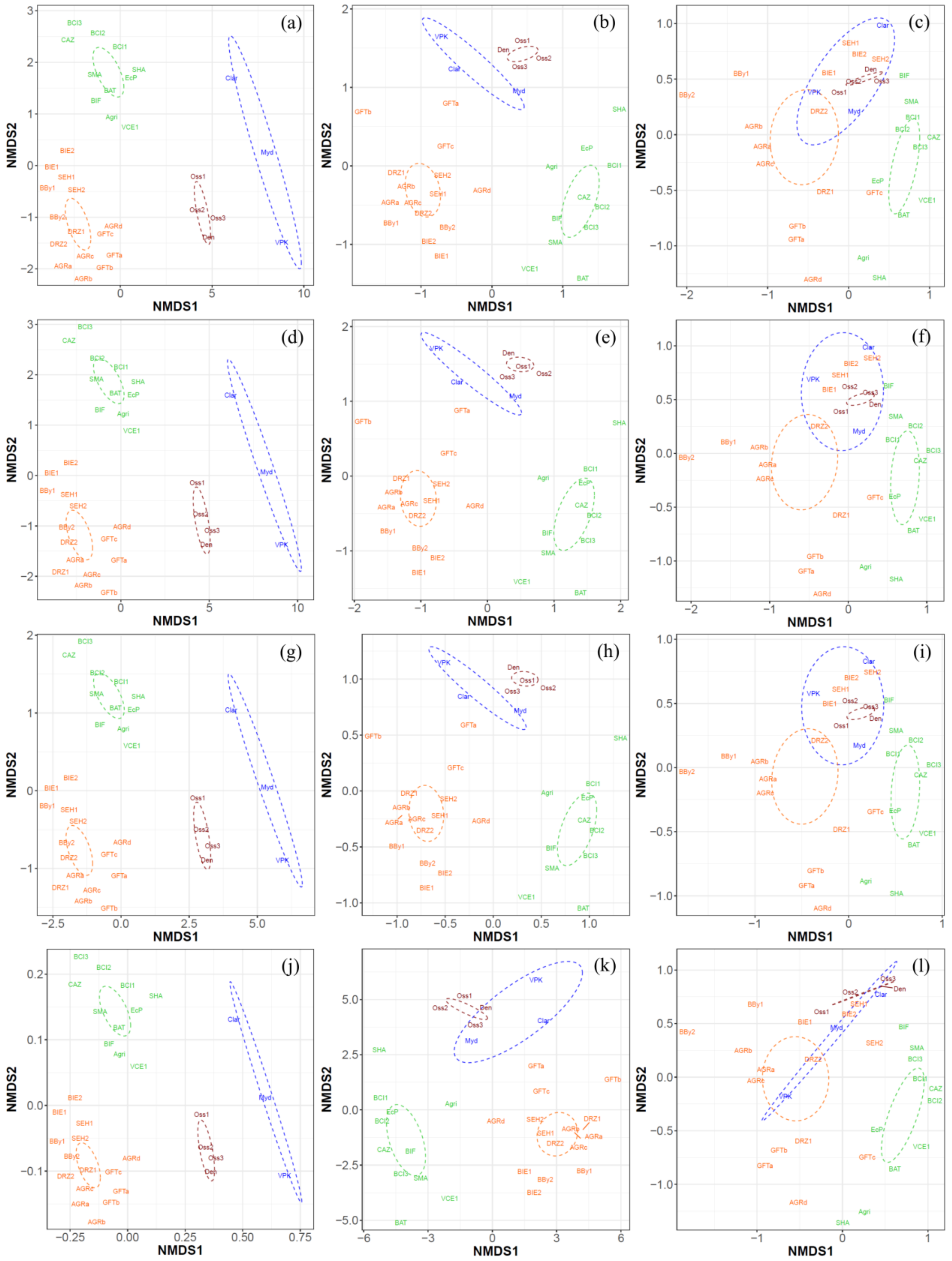

Figure S4 Non-metric multidimensional scaling (NMDS) analysis of the Chao (a-c), Jaccard (d-f), Kulczynski (g-i) and Mountford (j-l) distance measures for the Illumina (a, d, g and j), bacterial T-RFLP (b, e, h and k) and archaeal T-RFLP (c, f, i and l) data at OTU/TRF level. The four different clusters Cluster 1 (red), Cluster 2 (blue), Cluster 3 (green), and Cluster 4 (orange) are distinguished, and the ellipses represent the 95% value of the standard error of the average value for each cluster.

S7. Canonical correspondence analysis model results

Table S2 Overview of the results of the canonical correspondence model analysis (carried out with the *envfit* function, vegan package) by means of which the relation of the pH, temperature and total ammonia nitrogen (TAN) with the Illumina, bacterial TRFLP and archaeal TRFLP data was determined. Both the P-values (PERMANOVA) and R^2 (CCA model) values are shown.

P-values	Illumina	Bacterial TRFLP	Archaeal TRFLP
pH	0.0001	0.001	0.002
Temperature	0.0001	0.001	0.002
TAN	0.064	0.007	0.108
R^2 values			
pH	0.876	0.783	0.467
Temperature	0.858	0.776	0.518
TAN	0.830	0.898	0.459

References

1 McMurdie, P. J. & Holmes, S. Waste Not, Want Not: Why Rarefying Microbiome Data Is Inadmissible. *PLoS Comput. Biol.* **10**, 12, doi:10.1371/journal.pcbi.1003531 (2014).

2 Marzorati, M., Wittebolle, L., Boon, N., Daffonchio, D. & Verstraete, W. How to get more out of molecular fingerprints: practical tools for microbial ecology. *Environ. Microbiol.* **10**, 1571-1581, doi:10.1111/j.1462-2920.2008.01572.x (2008).

3 Lorenz, M. O. Methods of Measuring the Concentration of Wealth. *Publications of the American Statistical Association* **9**, 209-219, doi:10.2307/2276207 (1905).

4 Dejonghe, W., Boon, N., Seghers, D., Top, E. M. & Verstraete, W. Bioaugmentation of soils by increasing microbial richness: missing links. *Environ. Microbiol.* **3**, 649-657, doi:10.1046/j.1462-2920.2001.00236.x (2001).

5 Pareto, V. *Le Cours d'Economie Politique*. (Droz, 1897).
